# Abnormal Cerebrovascular Reactivity in Parkinson's Disease

**DOI:** 10.1155/2015/464052

**Published:** 2015-11-02

**Authors:** Kiran Prakash

**Affiliations:** Department of Physiology, Government Medical College & Hospital, Chandigarh 160030, India

We read with great interest the recent original article by Camargo et al. [1] titled “Abnormal Cerebrovascular Reactivity in Patients with Parkinson's Disease.” Authors observed that patients with Parkinson's disease (PD) have impaired cerebrovascular reactivity (CVR) as compared to those without orthostatic hypotension (OH) and to the control subjects. Also, authors have reported that there was not any significant difference between the Breath Holding Index (BHI) of control and patients with PD but without OH. This observation should be interpreted with caution because of important potential limitations of the study.

First, a major flaw was in the assessment of CVR. As it is established and authors have also mentioned that there is impairment in the cerebral autoregulatory capacity of patients with PD, proper corrections [2, 3] should be applied during CVR assessment. Hypercapnia per se can increase the systemic arterial blood pressure which can result in increased cerebral blood flow velocity and pseudo-high results of BHI. This may be the reason behind nonsignificant difference in the BHI values of control subjects and the subjects with PD without OH. Also, 54.5% of PD subjects and 27.3% of control subjects were found to have abnormal BHI. The cut-off value of BHI beyond which it is said to be abnormal has not been mentioned.

A second concern was regarding selection of control subjects. Controls were selected from healthy individuals recruited among the hospital staff and patient's relatives. Therefore, selection bias may be present in control selection as health concern may have influenced their participation.

The third flaw, also mentioned by the authors, was the small sample size. Therefore, nonsignificant associations reported in this study have to be interpreted with caution.

We congratulate Camargo et al. on this meticulous study exploring the cerebrovascular reactivity status in the patients with PD. However, clinicians and researchers should interpret this study with caution because of its shortcomings.
